# High blood glucose levels are associated with higher risk of colon cancer in men: a cohort study

**DOI:** 10.1186/s12885-017-3874-4

**Published:** 2017-12-12

**Authors:** Alexandra Vulcan, Jonas Manjer, Bodil Ohlsson

**Affiliations:** 1 Lund University, Skåne University Hospital, Department of Clinical Sciences, Division of Internal Medicine, Jan Waldenströms gata 14, 205 02 Malmö, Sweden; 2 Lund University, Skåne University Hospital, Department of Clinical Sciences, Division of Surgery, Inga Marie Nilssons gata 47, 205 02 Malmö, Sweden; 3 Lund University, Skåne University Hospital, Department of Clinical Sciences, Division of Internal Medicine, Jan Waldenströms gata 15, 205 02 Malmö, Sweden

**Keywords:** Blood glucose, Colorectal cancer (CRC), Homeostasis model assessment of insulin resistance (HOMA2-IR), Malmö diet and cancer study, Plasma insulin, Sex

## Abstract

**Background:**

High levels of blood glucose are thought to be associated with colorectal cancer (CRC) and hyperinsulinemia, an interstage in the development of CRC. The purpose of this study was to examine associations between incident CRC and blood glucose; plasma insulin; and the homeostasis model assessment for insulin resistance (HOMA2-IR), respectively, and to determine whether these associations were dependent on sex and cancer site.

**Methods:**

The Malmö Diet and Cancer cardiovascular cohort comprises 6103 individuals. During 81,781 person-years of follow-up, 145 cases of CRC were identified. The hazard ratio of measured blood glucose and plasma insulin and calculated HOMA2-IR were estimated with Cox proportional hazard regression.

**Results:**

An association was found between high levels of blood glucose and risk of CRC (HR: 1.72 for the highest compared with the lowest quartile; 95% CI: 1.05, 2.84; p_trend_ = 0.044), and colon cancer (HR: 1.70 for the highest compared with the lowest quartile; 95% CI: 0.87, 3.33; p_trend_ = 0.032). In men, an association was found between blood glucose and CRC (HR: 2.80 for the highest compared with the lowest quartile; 95% CI: 1.37, 5.70; p_trend_ = 0.001), and colon cancer (HR: 4.48 for the highest compared with the lowest quartile; 95% CI: 1.27, 15.84; p_trend_ = 0.007), but this was not found in women. No associations between plasma insulin, or HOMA2-IR, and CRC, were found.

**Conclusion:**

High levels of blood glucose in men are associated with risk of colon cancer. The findings contribute to facilitating to identify those most in need of prevention and screening.

## Background

Colorectal cancer (CRC) is one of the most common cancer forms in the Western world [1]. The increased incidence of CRC is associated with the increased incidence of lifestyle-related diseases, e.g., metabolic syndrome, overweight, obesity, and type 2 diabetes [2–4].

The latter diseases are characterized by hyperglycaemia, hyperinsulinemia, and insulin resistance [5]. The interaction between these diseases and CRC is being discussed, and molecular and etiological mechanisms are being sought. Hyperglycaemia might be associated with CRC [6], and the association may differ depending on different cancer sites and sex [4, 7]. Involvement of reactive oxygen species (ROS) and the accumulation of advanced glycation end products (AGE) are hypothesized to stimulate carcinogenic pathways [8–10]. Other proposals are that hyperinsulinemia drives the carcinogenic process through influence on the growth of cancer cells, stimulation of proliferation, decrease of apoptosis, and promotion of intestinal carcinogenesis [11]. Another hypothesis is that insulin resistance is responsible for the increased cancer risk. Although several studies have been conducted in the field, most of them have been case-control studies, and there has been discussion of whether further cohort studies are warranted [7].

## Method

The primary aim of this study was to examine the associations between incident CRC and blood glucose levels; plasma insulin levels; and homeostasis model assessment for insulin resistance (HOMA2-IR), respectively, and incident CRC. The secondary aim was to study whether the associations were dependent on sex and cancer site.

The study was approved by the Regional Ethical Review Board in Lund (50–91, 2013/804).

### Study population

The Malmö Diet and Cancer study (MDCS) is a large population-based study, conducted in the period 1991–1996 in Malmö, where all men and women, born between 1923 and 1950, were invited to participate. Altogether, 28,098 participants completed the baseline examinations after having given their written informed consent. The method of MDCS has previously been described by Manjer et al. [12]. The Malmö Diet and Cancer cardiovascular cohort (MDC-CC) comprises 6103 individuals randomly selected from MDCS. Out of the 6103 participants in MDC-CC, 5540 individuals accepted and were re-scheduled for blood sampling. 396 individuals did not leave a blood glucose and plasma insulin sample and were therefore excluded from the present study, leaving 5144 individuals (2117 men). From the remaining individuals, prevalent diabetes was found in 219 individuals (123 men), and prevalent CRC in 14 individuals (2 men), at baseline, all of whom were also excluded from the study, leaving 4910 individuals (1992 men).

### Cases of CRC

In the study, 145 cases of CRC (71 men) were identified from the Swedish Cancer Registry, of which 81 cases were colon cancer (37 men) and 64 were rectal cancer (34 men), during 81,781 person-years of follow-up. Follow-up time was defined as the time from the date of enrolment until the date of CRC diagnosis, death, migration or end of follow-up (31 December 2010), whichever came first. The mean follow-up was 16.7 ± 3.7 years.

### Plasma insulin, blood glucose and insulin resistance

In the MDC-CC all the blood and plasma samples were collected at the MDC-CC baseline by a trained nurse in the morning after 12 h of fasting, and plasma was separated and immediately frozen at −20 °C until analysed. Analyses were performed according to the clinical routines of the Department of Clinical Chemistry. Blood glucose was analysed using a routine hexokinase method. Insulin levels were measured in mIU/ml by a radioimmunoassay. The lowest limit for detection was 3 mlU/ml.

HOMA2-IR was calculated with the use of a HOMA2-IR calculator [13]. In the analysis of HOMA2-IR, extreme values of blood glucose (< 3.5 or >25 mmol/l) were excluded, as were extreme values of plasma insulin (< 3 or >57.5 mIU/ml), thus leaving 4451 individuals for analysis of HOMA2-IR. From the remaining individuals 135 cases of CRC, 76 cases colon cancer, and 59 cases of rectal cancer were found.

### Other variables

At MDCS baseline, age was obtained from personal identification numbers. Body Mass Index (BMI) was calculated from measured weight and length.

A self-administered structured questionnaire was used for the assessment of level of education, physical activity, alcohol consumption and smoking. The level of education was divided into four different categories: ≤ 8 years; 9–10 years; 11–13 years of education; and university degree. The subjects were asked to estimate their physical activity in terms of how many minutes per week they spent on 17 different activities. The duration was multiplied with an activity-specific intensity coefficient and an overall leisure-time physical activity score was created [14]. Alcohol intake was divided into four categories: zero; < 15 g/d for women and <20 g/d for men; 15–30 g/d for women and 20–40 g/d for men; and >30 g/d for women and >40 g/d for men. Smokers were divided into three categories: current smokers; ex-smokers; and non-smokers. Irregular smoking was counted as current smoking. The result was divided into quartiles.

Prevalent diabetes was determined by self-reported illness based on physician’s diagnosis or treatment with anti-diabetes medicine, or information from medical data registries indicating a date of diagnosis before inclusion in the MDC-CC. Incident diabetes diagnosis was obtained either from the Regional Diabetes 2000 register of Scania, the Malmö HbA1C register or the Swedish National Diabetes Register until 31st of December 2010. In the MDC-CC, 716 incident cases of diabetes (350 men) were found. In those with incident diabetes, 31 cases of incident CRC (22 men) were found.

### Statistics

The SPSS statistics (version 23; IBM Corporation, Armonk, NY, USA) was used for all statistical analyses.

Chi-square test was used when examining baseline categorical characteristics, and T-test when examining baseline continuous characteristics, in the cases and the non-cases.

The Cox proportional hazard regression model was used when estimating hazard ratios (HR) of incident CRC, colon cancer, and rectal cancer, depending on quartiles of blood glucose, plasma insulin and HOMA2-IR. The proportionality assumption was tested for all the adjustment factors with a Kaplan-Maier curve before analysis. Two models are presented: an unadjusted model, and a full model. The full model was adjusted for the background variables indicating a difference between the cases and the non-cases (*p* < 0.2), i.e. age, sex (when appropriate), BMI, and smoking status.

A test for interaction between sex and blood glucose; plasma insulin levels; and HOMA2-IR, respectively, with regard to CRC incidence was performed by adding a multiplicative variable (i.e. sex × blood glucose−/insulin-/HOMA2-IR quartiles (treated as continuous variables)) to the full model. If a significant interaction was found, subgroup analysis based on sex was performed.

In the sensitivity analysis, we excluded individuals with incident diabetes, and apart from that we made one additional model, full model glucose/insulin. The full model insulin was only used when estimating HR for quartiles of glucose and was adjusted for age, sex, BMI, smoking status, and insulin. The full model glucose was only used when estimating HR for quartiles of insulin and adjusted for age, sex, BMI, smoking status, and glucose. In addition, we excluded individuals with CRC diagnosis within two years of inclusion.

All tests were two-sided and statistical significance was assumed at *p* < 0.05.

## Results

Compared with the non-cases, the cases were older (Table 1), had larger waist circumference (86.9 ± 13.5 cm and 83.2 ± 12.6 cm, respectively, *p* = 0.019), and higher plasma insulin levels (9.6 ± 18.9 mIU/ml and 7.7 ± 7.1 mIU/ml, respectively, *p* = 0.012) at inclusion. More of the cases than non-cases developed diabetes during the follow-up (21.4% and 14.6%, respectively).

**Table 1:** Baseline characteristics of cases and non-cases in the Malmö Diet and Cancer Study cardiovascular cohort

	Cases *n* = 145 (%)	Non-cases *n* = 4765 (%)	*p*-value^a^
Sex			0.037
- Men	71 (49.0)	1921 (40.1)	
- Women	74 (51.0)	2844 (59.9)	
Age (years)	61.3 ± 7.0	57.4 ± 5.9	<0.001
Age quartiles			<0.001
45.8–52.4 years	22 (15.2)	1205 (25.3)	
52.4–57.8 years	26 (17.9)	1202 (25.2)	
57.8–62.7 years	34 (23.4)	1194 (25.1)	
62.7–68.1 years	63 (43.5)	1164 (24.4)	
Body Mass Index (kg/ m^2^)	26.2 ± 3.9	25.6 ± 3.9	0.032
Body Mass Index ≤25 kg/m^2^ >			0.155
- ≤ 25 kg/m^2^	63 (43.4)	2356 (49.4)	
- > 25 kg/m^2^	82 (56.6)	2409 (50.6)	
Education			0.732
- > 10 years	42 (29.0)	1335 (28.0)	
- ≤ 10 years	103 (71.0)	3425 (71.9)	
- Missing	0 (0)	5 (0.1)	
Physical activity			0.439
- High^c^	38 (27.3)	1182 (24.5)	
- Lower^c^	105 (71.3)	3555 (74,9)	
- Missing	2 (1.4)	28 (0.6)	
Alcohol intake			0.832
- Zero	26 (17.9)	742 (15.6)	
- < 15 g/d for women and <20 g/d for men	91 (62.8)	3088 (64.8)	
- 15–30 g/d for women and 20–40 g/d for men	21 (14.5)	757 (15.9)	
- > 30 g/d for women and >40 g/d for men	7 (4.8)	173 (3.6)	
- Missing	0 (0)	5 (0.1)	
Smoking			0.023
- Current	31 (21.4)	1307 (27.4)	
- Ex	65 (44.8)	1556 (32.7)	
- Never	49 (33.8)	1900 (39.9)	
- Missing	0 (0)	2 (0.04)	

^a^Calculated with Chi-square test for categorical variables and with T-test for the continuous variables

Physical activity was defined as high when in the highest quartile of the whole group, and lower when in the three lower quartiles [14]

Values are number of individuals and percentage or mean and standard deviation. *p* < 0.05 was considered statistically significant

### Blood glucose levels and colorectal, colon, or rectal cancer

An association between high levels of blood glucose and risk of CRC and colon cancer was found (HR: 1.72 for the highest compared with the lowest quartile; 95% CI: 1.05, 2.84, p for trend = 0.044) and (HR: 1.70 for the highest compared with the lowest quartile; 95% CI: 0.87, 3.33, p for trend = 0.032) (Table 2), respectively. With regard to blood glucose levels, an interaction between sex was found in CRC (*p* = 0.013) and in colon cancer (*p* = 0.032), but not in rectal cancer (*p* = 0.130). A significant association between high levels of blood glucose and CRC was found in men (HR: 2.80 for the highest compared with the lowest quartile; 95% CI: 1.37, 5.70; p for trend = 0.001), but not in women (HR: 1.02 for the highest compared with the lowest quartile; 95% CI: 0.53, 1.95; p for trend = 0.739) (Table 3). Also, a significant association was found between high levels of blood glucose and colon cancer in men (HR: 4.23 for the highest compared with the lowest quartile; 95% CI: 1.46, 13.44; p for trend = 0.002), but not in women (HR: 1.01 for the highest compared with the lowest quartile; 95% CI: 0.44, 2.34; p for trend = 0.878) (Table 4).

**Table 2:** Hazard ratio (HR) of incident colorectal-, colon, and rectal cancer associated with blood glucose, plasma insulin and Homeostasis Model Assessment for Insulin Resistance (HOMA2-IR) in the Malmö Diet and Cancer Study cardiovascular cohort

			Colorectal cancer				Colon cancer						Rectal cancer			
			Crude model		Full model			Crude model		Full model			Crude model		Full model	
Quartiles	Min-max	Cases/person-years	HR	95% CI	HR	95% CI	Cases/person-years	HR	95% CI	HR	95% CI	Cases/person-years	HR	95% CI	HR	95% CI
Blood glucose (mmol/l) *n* = 4910																
1	3.3–4.5	25/18381	1.00		1.00		14/18246	1.00		1.00		11/18215	1.00		1.00	
2	4.6–4.8	30/21325	1.04	0.61, 1.76	0.94	0.55, 1.60	13/21193	0.80	0.38, 1.70	0.71	0.33, 1.51	17/21197	1.33	0.62, 2.83	1.21	0.56, 2.60
3	4.9–5.2	34/22388	1.12	0.67, 1.87	0.99	0.59, 1.68	22/22277	1.29	0.66, 2.52	1.11	0.56, 2.21	12/22163	0.89	0.39, 2.03	0.78	0.34, 1.81
4	5.3–16.8	56/19687	2.11	1.31, 3.37	1.72	1.05, 2.84	32/19476	2.17	1.15, 4.06	1.70	0.87, 3.33	24/19360	2.05	1.00, 4.18	1.62	0.75, 3.45
p for trend			0.003		0.044			0.005		0.032			0.165		0.530	
p for interaction between sex and blood glucose					0.013					0.032					0.130	
Plasma insulin (mIU/l) n = 4910																
1	<3–4	33/23177	1.00		1.00		22/2047	1.00		1.00		11/22922	1.00		1.00	
2	5–6	32/19306	1.16	0.72, 1.89	1.09	0.67, 1.78	17/19190	0.93	0.49, 1.75	0.85	0.45, 1.60	15/19154	1.63	0.75, 3.55	1.58	0.72, 3.45
3	7–8	33/16926	1.37	0.84, 2.22	1.25	0.76, 2.04	20/16800	1.25	0.68, 2.29	1.09	0.59, 2.03	13/16710	1.62	0.72, 3.06	1.55	0.69, 3.52
4	9–224	47/22372	1.48	0.95, 2.32	1.21	0.75, 1.96	22/22154	1.05	0.58, 1.89	0.80	0.42, 1.50	25/21149	2.35	1.16, 4.78	2.12	0.99, 4.53
p for trend			0.064		0.411			0.645		0.635			0.024		0.078	
p for interaction between sex and plasma insulin					0.142					0.350					0.316	
HOMA2-IR *n* = 4451																
1	0.3–0.5	23/13702	1.00		1.00		16/13623	1.00		1.00		7/13514	1.00		1.00	
2	0.5–0.8	30/20111	0.98	0.59, 1.62	0.91	0.55, 1.50	16/19996	0.80	0.42, 1.53	0.72	0.37, 1.38	14/19966	1.33	0.60, 2.92	1.27	0.58, 2.82
3	0.8–1.2	37/20527	1.19	0.74, 1.91	1.06	0.65, 1.72	22/20387	1.08	0.59, 1.96	0.92	0.50, 1.70	15/20288	1.40	0.64, 3.04	1.33	0.60, 2.95
4	1.2–7.3	45/19776	1.51	0.96, 2.37	1.23	0.75, 2.01	22/19574	1.13	0.62, 2.06	0.84	0.44, 1.33	23/19553	2.23	1.09, 4.57	2.03	0.93, 4.42
p for trend			0.066		0.429			0.527		0.733			0.040		0.116	
p for interaction between sex and HOMA2-IR					0.099					0.211					0.789	

Full model: Calculated with the Cox proportional hazard regression model**.** Adjusted for sex (man, woman), age (quartiles of age), BMI (≤ 25 kg/m^2^, > 25 kg/m^2^), and smoking (current, ex or never). Values are quartile ranges, hazard ratios (HR), and 95% confidence intervals (CI)

**Table 3:** Hazard ratio (HR) of incident colorectal- and colon cancer associated with blood glucose for men and women in the Malmö Diet and Cancer Study cardiovascular cohort

		Colorectal cancer n_men_ = 1992 n_women_ = 2981					Colon cancer n_men_ = 1958 n_women_ = 2888				
			Crude model		Full model			Crude model		Full model	
Quartiles of glucose	Min-max (mIU/l)	Cases/person-years	HR	95% CI	HR	95% CI	Cases/person-years	HR	95% CI	HR	95% CI
Men											
1	3.3–4.5	5/4400	1.00		1.00		2/4358	1.00		1.00	
2	4.6–4.8	10/7276	1.28	0.57, 2.90	1.25	0.55, 2.85	4/7216	2.05	0.50, 7.01	1.95	0.57, 6.70
3	4.9–5.2	16/9835	2.10	0.99, 4.45	2.03	0.95, 4.32	9/9767	2.28	0.67, 7.79	2–08	0.60, 7.17
4	5.3–16.8	40/10853	3.08	1.54, 6.15	2.80	1.37, 5.70	22/19679	5.40	1.84, 15.87	4.23	1.46, 13.44
p for trend			<0.001		0.001			0.001		0.002	
Women											
1	3.4–4.5	20/13981	1.00		1.00		12/13888	1.00		1.00	
2	4.6–4.8	20/14048	1.24	0.64, 2.38	1.21	0.62, 2.34	9/13977	0.77	0.29, 2.05	0.73	0.27, 1.96
3	4.9–5.2	18/12553	0.89	0.47, 1.66	0.82	0.44, 1.55	13/12510	1.08	0.50, 2.34	0.96	0.45, 2.09
4	5.3–12.2	16/8834	1.16	0.62, 2.17	1.02	0.53, 1.95	10/8797	1.22	0.54, 2.71	1.01	0.44, 2.34
			0.908		0.739			0.562		0.878	

Full model: Calculated with the Cox proportional hazard regression model**.** Adjusted for age (quartiles of age), BMI (≤ 25 kg/m^2^, > 25 kg/m^2^), and smoking (current, ex or never)

Values are quartile ranges, hazard ratios (HR), and 95% confidence intervals (CI)

**Table 4:** Hazard ratio (HR) of incident colorectal cancer associated with Homeostasis Model Assessment for Insulin Resistance (HOMA2-IR) for men and women in the Malmö Diet and Cancer Study cardiovascular cohort

			Unadjusted model		Full model	
Quartiles of HOMA2-IR	Min-max (mIU/l)	Cases/person-years	HR	95% CI	HR	95% CI
Men (*n* = 1824)						
1	0.35–0.63	6/3938	1.00		1.00	
2	0.64–0.90	9/7615	0.66	0.30, 1.47	0.64	0.29, 1.44
3	0.91–1.28	19/8618	1.35	0.69, 2.63	1.29	0.65, 2.56
4	1.29–6.85	30/9434	1.80	0.95, 3.41	1.62	0.81, 3.25
p for trend			0.042		0.132	
Women (*n* = 2627)						
1	0.34–0.51	17/9764	1.00		1.00	
2	0.52–0.77	21/12496	1.26	0.67, 2.37	1.11	0.59, 2.09
3	0.78–1.06	18/11908	1.15	0.60, 2.18	0.97	0.50, 1.89
4	1.07–7.30	15/10309	0.97	0.49, 1.89	0.77	0.37, 1.59
p for trend			0.760		0.779	

HR was calculated with the Cox proportional hazard regression model

Unadjusted model: No adjustments made

Full model: Adjusted for age (quartiles of age), BMI (≤ 25 kg/m^2^, > 25 kg/m^2^), and smoking (current, ex or never)

Values are quartile ranges, hazard ratios (HR), and 95% confidence intervals (CI)

### Plasma insulin levels and colorectal, colon, or rectal cancer

No significant associations were found between plasma insulin levels and CRC, colon, or rectal cancer (Table 2). No interaction between sex and plasma insulin levels was found in CRC (*p* = 0.142), colon cancer (*p* = 0.358), or in rectal cancer (*p* = 0.280).

### HOMA2-IR and colorectal, colon, or rectal cancer

No significant associations between HOMA2-IR and CRC, colon, or rectal cancer were found in the full model (Table 2). There was a borderline interaction between sex and HOMA2-IR in CRC (*p* = 0.099), but not in colon cancer (*p* = 0.211), or rectal cancer (*p* = 0.789). The associations between HOMA2-IR and CRC did not reach statistical significance in men (HR: 1.62 for the highest compared with the lowest quartile; 95% CI: 0.81, 3.25; p for trend = 0.132), or in women (HR: 0.77 for the highest compared with the lowest quartile; 95% CI: 0.37, 1.59; p for trend = 0.779) (Table 4).

### Sensitivity analysis

After excluding those with incident diabetes, the association between blood glucose and CRC was no longer significant (HR: 1.74 for the highest compared with the lowest quartile; 95% CI: 1.00, 3.07, p for trend = 0.134). The same was true for the association between blood glucose and colon cancer (HR: 1.71 for the highest compared with the lowest quartile; 95% CI: 0.80, 3.68, p for trend = 0.128). In men, the association between blood glucose and colon cancer had borderline significance (HR: 3.47 for the highest compared with the lowest quartile; 95% CI: 0.88, 13.74, p for trend = 0.099).

In excluding those with diagnosis of CRC within two years from inclusion in the study (13 individuals, where of eight were men), the association between blood glucose and CRC had borderline significance (HR: 1.71 for the highest compared with the lowest quartile; 95% CI: 1.01, 2.89, p for trend = 0.090), and the risk estimate for the association between blood glucose and rectal cancer in men increased (HR: 5.20 for the highest compared with the lowest quartile; 95% CI: 1.47, 18.43, p for trend = 0.006).

In additionally adjusting for insulin in the full model for glucose, neither the risk estimate, nor the significance substantially changed. Nor did they substantially change when additionally adjusting for glucose in the full model for insulin (data not shown).

## Discussion

The results from the present study indicate that high levels of blood glucose are associated with risk of CRC, more specifically in men. No associations were found for insulin or HOMA2-IR and risk of CRC.

Our results are in agreement with other studies, which also found an association between high glucose levels and CRC, with significant associations in men, but not in women, and in colon cancer, but not in rectal cancer [7]. Glucose induces expressions of Amphiregulin, through transcriptional regulation of the MAX-like protein X [15], suggesting that one part of the tumorigenesis in CRC might be glycolysis [16]. Furthermore, hyperglycaemia gives energy to malignant cells for their proliferation [16], and thus favours cancer growth and neoangiogenesis. Since there can be an insulin-independent glucose uptake in cancer cells, this may have an impact on the association between glucose, insulin and risk of CRC.

In hyperglycaemia, the cells’ production of reactive oxygen intermediates increases, and is therefore speculated to be a part of the induction of apoptosis in endothelial cells [17]. Chronic inflammation, present for example during hyperglycaemia, leads to imbalance between production and restoration of ROS, leading to oxidative stress within the target tissue, which may damage DNA and reduce DNA repair [18].

Glucose and insulin levels are closely linked, which makes it difficult to separate the association, although they influence the cancer development through different pathways. Insulin stimulates the proliferation of cells partly through binding insulin to insulin- or insulin-like growth factor 1 (IGF-1) receptors, and partly through inhibition of IGF-binding proteins, thus increasing the availability for IGF-1 to bind to IGF-1 receptors [19]. Circulating IGF-1 is thought to increase the risk of CRC [20]. In a meta-analysis by Xu et al. [7], an association between plasma insulin levels and risk of CRC was found in case-cohort studies, but not in cohort studies, in line with the present cohort study.

Insulin resistance, hyperinsulinemia, and hyperglycaemia are important factors in metabolic syndrome. When insulin resistance develops in type 2 diabetes, the pancreatic insulin secretion is increased in compensation [21]. It is speculated that excess of insulin might enhance colonic epithelial activity and induce the formation of aberrant cryptic foci [22]. Insulin resistance affects the metabolic pathways, over-stimulates the mitogenic pathways and stimulates cell proliferation [23]. However, in the present study no association was found between HOMA2-IR and CRC, but this might be due to the small number of cases in the analysis.

In the present study, more of the cases than the non-cases developed diabetes. They also had a larger waist circumference. Some research has suggested that the association between diabetes and CRC may not be causal; rather the two diseases just share the same preconditions, such as obesity [24]. This might be a reason why only high glucose levels, and not insulin and insulin resistance, show risk association. Metformin reduces insulin resistance and improves glycaemic control [25]. High intake of metformin seems to be protective against CRC [26]. The hypothesis is that metformin slows the progression and growth of the tumour [27]. In the present study, it was not possible to determine whether metformin was used between inclusion and the end of study, and this might have affected the results. However, in excluding incident diabetes in the sensitivity analysis, the effect on cancer development might have been avoided, but also excluding those with higher blood glucose, and decreasing the possible number of cases in the analysis.

As seen in our previous and present study on the MDCS, associations with CRC might not be straightforward, but may be dependent on sex and cancer site [28]. CRC may differ in nature depending on whether it is a distal or proximal CRC, with more micro instability in the distal CRC and more chromosomal instability in the proximal cancer [29]. Whether this difference can explain the variations in associations between rectal and colon cancer remains to be determined. There is a difference in the DNA methylation based on sex. An association between altered expressions and insulin secretion in the human pancreatic islet has been found, where women have a higher glucose-induced insulin secretion [30]. Oestrogen also plays an important role in the glucose metabolism in the body. Oestrogen facilitates the transport of glucose to the brain and promotes neural aerobic glycolysis [31]. In men, 5-DHEA, an adrenal steroid hormone which modulates glucose uptake, is more elevated than in women [32]. As there are differences in the glucose metabolism for men and women, high glucose levels may affect the risk differently, as seen in the present study.

The strength of the present research is that it is a cohort study with a long follow-up. A limitation of the study is the small number of cases, and it cannot be excluded that some of the stratified analyses may be underpowered. Taking family history of CRC or inflammatory bowel disease into account in our analyses would have been valuable, since they are contributing factors in the development of CRC [33], but information on these risk factors was missing, as was information on family history of diabetes and blood lipid profile. Even if adjustments for possible confounders and known risk factors were made, the occurrence of some residual confounding cannot be excluded.

## Conclusion

High levels of blood glucose are associated with risk of CRC, mainly with colon cancer in men. The findings contribute to facilitating to identify those most in need of prevention and screening.

## Abbreviations

AGE: Advanced Glycation End products
BMI: Body Mass Index
HOMA2-IR: Homeostasis Model Assessment for Insulin Resistance
IGF-1: Insulin-like Growth Factor 1
MDC-CC: The Cardiovascular Cohort in the Malmö Diet and Cancer study
MDCS: Malmö Diet and Cancer study
ROS: Reactive Oxygen Species
